# The Role of Ultrahigh Resolution Fourier Transform Mass Spectrometry (FT-MS) in Astrobiology-Related Research: Analysis of Meteorites and Tholins

**DOI:** 10.3390/ijms17040439

**Published:** 2016-03-24

**Authors:** Árpád Somogyi, Roland Thissen, Francois-Régis Orthous-Daunay, Véronique Vuitton

**Affiliations:** 1 Campus Chemical Instrument Center, Mass Spectrometry and Proteomics Laboratory, Ohio State University, Columbus, OH 43210, USA; 2 Université Grenoble Alpes, CNRS, IPAG, Grenoble F-38000, France; roland.thissen@univ-grenoble-alpes.fr (R.T.); frod@univ-grenoble-alpes.fr (F.-R.O.-D.); veronique.vuitton@univ-grenoble-alpes.fr (V.V.)

**Keywords:** origin of life, prebiotic material, ultrahigh resolution mass spectrometry, Fourier transform (FT), Orbitrap, ion cyclotron resonance (ICR), chemical compositions, isomeric structures

## Abstract

It is an important but also a challenging analytical problem to understand the chemical composition and structure of prebiotic organic matter that is present in extraterrestrial materials. Its formation, evolution and content in the building blocks (“seeds”) for more complex molecules, such as proteins and DNA, are key questions in the field of exobiology. Ultrahigh resolution mass spectrometry is one of the best analytical techniques that can be applied because it provides reliable information on the chemical composition and structure of individual components of complex organic mixtures. Prebiotic organic material is delivered to Earth by meteorites or generated in laboratories in simulation (model) experiments that mimic space or atmospheric conditions. Recent representative examples for ultrahigh resolution mass spectrometry studies using Fourier-transform (FT) mass spectrometers such as Orbitrap and ion cyclotron resonance (ICR) mass spectrometers are shown and discussed in the present article, including: (i) the analysis of organic matter of meteorites; (ii) modeling atmospheric processes in ICR cells; and (iii) the structural analysis of laboratory made tholins that might be present in the atmosphere and surface of Saturn’s largest moon, Titan.

## 1. Introduction

The chemical composition and structure of prebiotic materials that are building blocks of more complex, biologically important molecules, including peptides, lipids, proteins, DNA, *etc.*, provide important information on their formation. Obviously, the creation of heavy elements (*i.e.*, heavier than He) is prerequisite for the formation of very small “pre-prebiotic” molecules and radicals, such as H_2_O, CO, CH_4_, CH_2_=O, N_2_, HCN molecules and OH, CN radicals/anions, *etc.* (for a recent review of the evolution of elements and organic matter in space, see [1]).

The early ingredients for organic matter were formed in interstellar clouds. Since the 1960s, microwave (MW) spectroscopy has provided strong evidence for the existence of these small molecules in interstellar clouds [2]. Almost 130 such molecules are known and identified by MW spectroscopy. Recently, high level quantum mechanical calculations allow further refinement of the observed spectra confirming these species more reliably (see, e.g., Reference [3]). The concentration of these molecules in interstellar clouds, however, is too small and the temperature is too cold to form more complex prebiotic molecules in large number and with reasonable rates (even on the cosmic timeframe). Nevertheless, shock waves generated by nearby supernovae swings the pendulum of molecular synthesis far away from the (cold) equilibrium and thus complex chemistry does occur. Over time, complex molecules such as amino acids, most notably glycine are formed and they can be delivered on the surfaces of rocky planets, ranging in size from nanometric dust to space rocks (asteroids), which can later be captured by the gravitational field of (newly formed) stars. It is well known that amino acids and other prebiotic molecules have been detected in meteorites that fell on Earth. From an astrobiological point of view, meteorites are of particular importance because we can study their chemical compositions in Earth based laboratories. Among other methods, such as nuclear magnetic resonance (NMR), ultrahigh resolution mass spectrometry (UHRMS) is a very effective tool to study the chemical composition of *individual* chemical compounds delivered from space. Below we will mention some of these studies.

The alternative to sample delivery by meteorites is to send laboratories to places where complex organic molecules can be formed. Unfortunately, present technologies prevent us from launching analytical instruments beyond the Solar System so we are restricted to a limited number of Sun bound “laboratories”. Potential candidates include planets and/or moons with atmospheres. Besides Earth, Venus, Mars, and Pluto, only Saturn’s largest moon, Titan has an atmosphere in the Solar System (in fact, a very thick one) consisting mostly of N_2_, with 2% CH_4_, 0.4% H_2_ and trace amounts (ppm or ppb) of oxygen containing species such as CO. Analytical instruments, including mass spectrometers, have been sent to several Solar System bodies, including planets, comets, and moons. The success of the Cassini–Huygens mission provided us data on charged species in the atmosphere of Titan by using the “on-board” ion-neutral mass spectrometer (INMS) [4,5], Cassini plasma spectrometer (CAPS-IBS) [6], and the electron spectrometer (CAPS-ELS) [7,8].

An appealing and much less expensive alternative to sending spacecrafts (mass spectrometers) to celestial bodies is using Earth-linked laboratories to mimic, e.g., atmospheric processes and analyze their products. Although this approach does not give direct information on the “*in situ*” environment, it is flexible in the sense that we can easily change experimental conditions and we can use different instrumentations to analyze the outcome in great details. The products of these model reactions performed on Earth are very complex mixtures so mass spectrometry, especially ultrahigh resolution mass spectrometry occasionally coupled with separation techniques, such as gas or liquid chromatography (GC and LC, respectively) is a perfect tool to obtain information on chemical compositions and structures.

The research area on prebiotic materials is so broad and diversified that it cannot be adequately reviewed in a paper with limited length. Therefore, in the present paper, we show and discuss a few demonstrative examples of how useful ultrahigh resolution mass spectrometry (UHRMS) is in these astrobiologically related studies. We focus mostly on results obtained by the two best “performers” in terms of resolving power, both Fourier Transform: the FT-Orbitrap and FT-ICR mass spectrometers. The purpose of this paper is not to provide a comprehensive review of all mass spectrometry studies in the astrobiological field. Nevertheless, for detail-oriented readers, we recommend a recently published review on Titan tholins [9] and also the references throughout the paper. We will focus on the UHRMS analysis of: (i) organic contents of meteorites; (ii) the immediate, small molecule products of laboratory modeling of Titan’s atmospheric reactions; and (iii) the structural investigation of higher molecular weight C_*x*_H_*y*_N_*z*_ (>60 Da) species and their hydrolysis products.

## 2. Results and Discussion

### 2.1. Determination of Organic Components in Meteorites by FT-MS

Obviously, from an astrobiological point of view, organic compounds containing C, H, N, O, S, and P, are of primary astrobiological interest [10,11,12,13,14]. Soluble organic matter (SOM) can be extracted from powdered meteorite by common solvents (e.g., methanol or dichloromethane) and these extracts can be ionized by electrospray ionization in both the positive and negative ion modes. Nevertheless, inorganic content, such as the presence of mass deficient MgSO_4_, Na salts, and minerals can also be identified in otherwise organic rich chondrites [10].

**Figure 1:**
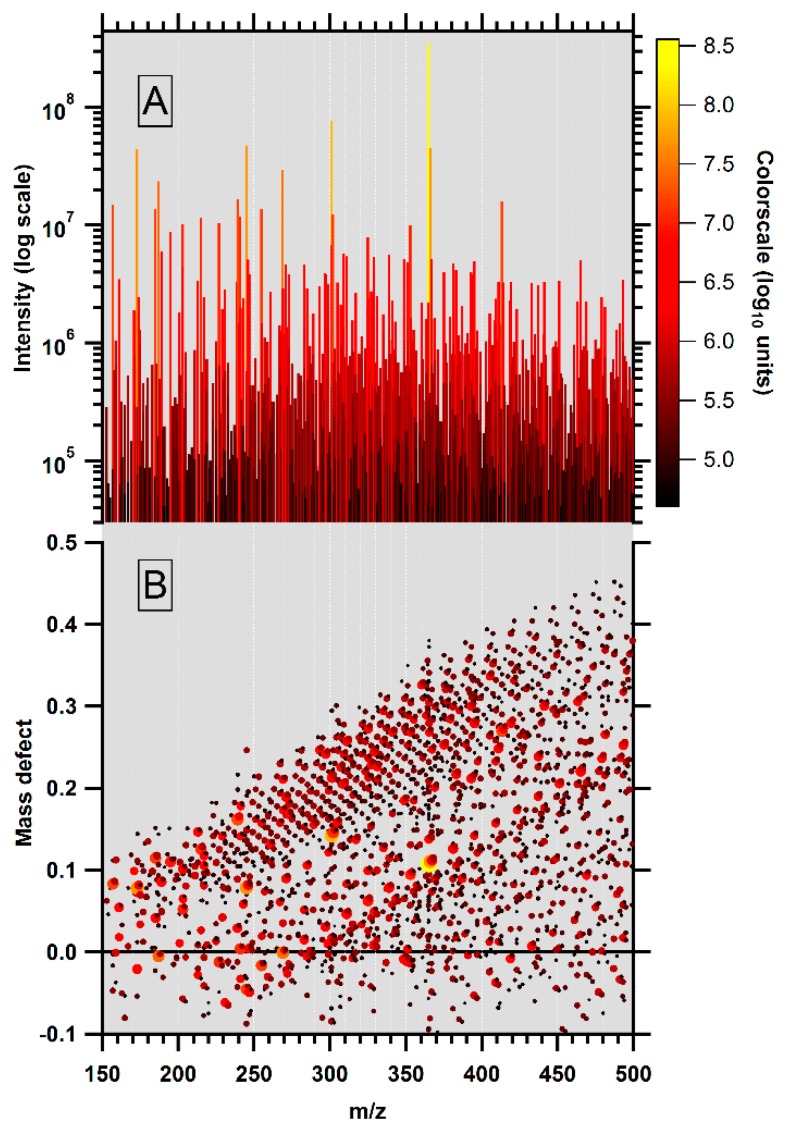
Martian sample organic matter extracted from ~20 mg of whole rock after 24 h maceration in 4 mL of methanol/toluene (1:2) at room temperature followed by storage in a dark place. (**A**) ESI produced positively charged mass spectrum. More than 2000 masses are detected in four decades in the 150–500 Da range. Average density in mass is 6.2 ions per Da. Neither a remarkable envelope nor peculiar organization can be seen. Colors indicate intensities on a logarithmic scale; (**B**) MDvEM representation of the mass spectrum. Each point represents one mass each. Mass defect is the difference between the mass coordinate and its closest integer so the value spans periodically and linearly within the −0.5 to +0.5 *m/z* range along the mass coordinate. This gives a modular space (like hours and minutes on a watch) where repetitive occurrences of mass difference draw lines. The visually remarkable alignment of points here is due to the CH_2_ pattern repetition. Only singly charged ions are considered.

For illustration, we present here some of our recent results using the ultrahigh resolution LTQ-Orbitrap (Grenoble, France) to analyze the soluble organic matter from a Martian meteorite (Figure 1). Small meteor rocks were ground and a methanol:toluene (1:2) solvent mixture was used for extraction. After centrifugation, direct infusion ESI was used in the positive ion mode to generate ions. The spectrum in Figure 1A contains a large number of peaks showing hundreds of components. Note that the vertical (intensity) axis is logarithmic in Figure 1A so the intensity range shown is 1–50,000. A mass defect *versus* exact mass (MDvEM) plot (for appropriate references, see Materials and Methods below) of the same and intricate MS is shown in Figure 1B. MDvEM analysis reveals immediately the very high degree of organization in the datasets, with obvious series in CH_2_, but also addition of minerals, due to the interaction within the complex matrix (Figure 1B). Intensity dimension is color coded while the vertical axis plots the difference of one exact mass to the nearest integer. Repetition of stoichiometric patterns generates lines in this diagram. Here, the most visible oblique lines are due to the multiple addition of CH_2_.

The insoluble organic material (IOM) consists mostly of unsaturated polycyclic macromolecules that are difficult to ionize by ESI. Thus, the availability of dual MALDI/ESI sources (such as on the Bruker Apex Qh or SolarixR instruments) is incredibly useful. We detected organic molecules (ions) using simply laser desorption ionization (LDI) of Orgueil. In general, our results confirmed that ESI and LDI are complementary ionization techniques that probe different parts (organic content) of chondritic meteorites [10] (data not shown, but see other LDI results below).

Recent LTQ-Orbitrap studies also revealed that carbonaceous meteorites (such as Murchison and Lonewolf Nunatks 94,103 meteorites) contain a wide range extraterrestrial nucleobasis. [11] Tandem MS/MS fragmentation and comparison with reference standard provided unambiguous proof for the presence of purine, adenine, 2,6-diaminopurine and 6,8-diaminopurine that can be considered as components of an “extended genetic alphabet”.

Note here that although FT-MS measurements (Orbitrap or ICR) provide reliable chemical compositions, it is difficult to distinguish between structural isomers based solely on (ultrahigh resolution) tandem MS/MS fragmentation. Other techniques, such as nuclear magnetic resonance (NMR) are extremely useful and improve the information content of analysis. A world leader in these combinative studies is a group led by Schmitt–Kopplin in the Helmholz–Zentrum in Munich, Germany [12,13]. Their recent studies include the one on Soltmany ordinary chondrite [12] and Murchison meteorite [13].

For correctness, we also note that conventional low resolution MS coupled with chromatography is still justified to use and provides useful information on organic content. An interesting recent example is a study when NWA, Gold Basin, Orgueil, and Al Haggounia meteorite powders were used to catalyze synthesis of nucleosides and other prebiotic compounds from formamide under proton irradiation [14]. Under these hostile conditions, about 30 different organic compounds have been detected by GC-MS. Because most of these compounds are not volatile enough derivatization was necessary before the analysis [14].

Based on the literature and our own experience, we can conclude that ultrahigh resolution MS (and tandem MS/MS) experiments are important to reveal detailed chemical information on extraterrestrial organic matter delivered to the Earth by meteorites. Although ESI is definitely a powerful ionization method, for the analysis of certain insoluble organic material we strongly recommend the use of laser desorption ionization (LDI) as a complementary ionization method (for other examples, related to laboratory “tholins”, see below).

### 2.2. Modeling Reactions in Titan’s Atmosphere and Analyzing the Products by FT-MS

Atmospheric processes have long been known to generate organic molecules. As mentioned in the Introduction, Titan’s atmosphere is well known due to the successful Cassini–Huygens mission. Specifically, the INMS [4,5] mass spectrometer detected hydrocarbons and N-containing species up to *m*/*z* 100. Due to low (unit) resolution, however, the precise assignments are ambiguous. Heavier positively and negatively charged ions (with higher *m*/*z* up to several hundreds or even thousands) were detected by the CAPS-IBS [6] and CAPS-ELS [7,8] mass spectrometers, respectively. This is very important *in situ* information, but again, due to low resolution, it prevents reliable identification of the detected ions. Moreover, it is not clear how much and by what mechanism nitrogen is incorporated in Titan’s organic aerosols, *i.e.*, the formation mechanisms of astrobiologically important organics are not fully understood.

To improve our understanding, in line with experiments, theoretical modeling of the kinetics of Titan’s atmospheric processes is of primary importance. These studies are out of the scope of the current paper but we refer here to the work of Vuitton, Lavvas, Yelle, and others [15,16,17,18,19].

Obviously, for generating ion–molecule (or even molecule–molecule) interactions an energy source is required. On Titan (and, in general, other Solar System bodies with atmosphere), one of these sources are high enough energy photons in the UV range that are capable of ionizing/dissociating N_2_ (and/or CH_4_). Using tunable synchrotron radiation at the Advanced Light Source, Imanaka and Smith recently demonstrated the first evidence of nitrogenated organic aerosol production by extreme ultraviolet–vacuum ultraviolet irradiation of a N_2_/CH_4_ gas mixture [20]. The products of these UV-induced reactions were then analyzed by a low resolution quadrupole mass analyzer and by ultrahigh-mass-resolution FT-ICR (Bruker 9.4 T Apex Qh, Bruker, Billarica, MA, USA) mass spectrometry with LDI. The N_2_/CH_4_ photolytic solid products at 60 and 82.5 nm irradiation wavelengths indicate the predominance of highly nitrogenated compounds. ^15^N labeling predicted that short lived nitrogen containing reactive species, such as the HC_2_N radical might play a role in the formation of C_*x*_H_*y*_N_*z*_ species. We note, however, that isotope labeling of the gas mixtures with ^13^C clearly indicated the presence of unsaturated hydrocarbons at 60 nm irradiation [20].

FT-ICR cells can be used not only to detect large number of ions with high resolution and *m/z* precision but also to perform ion-molecule reactions in the cell. Maybe the simplest example is hydrogen/deuterium exchange (HDX) reactions of isomeric ions [21]. Different HDX rates indicate structural isomers of isomeric ions. In some cases, reaction products are also different (see, e.g., ND_3_ addition to b_n_ peptide fragment ions, when *n* > 5 [22]).

A novel experiment relevant to modeling Titan’s atmospheric chemistry has been performed inside the cell of a FT-ICR mass spectrometer [23]. The ICR cell served as a “reaction chamber” into which gas mixtures representative of Titan’s atmospheric composition were injected. These gas mixtures were then irradiated with extreme ultraviolet radiation from the Elettra synchrotron. The influence of various gas mixtures and irradiation frequencies were studied on the ion evolution. This approach allowed the authors to compare the theoretical model predictions [15] to the laboratory experiments and to test for missing and/or poorly constrained pathways. Overall, the comparison between observed and modeled ion intensities validates the kinetic model. Unfortunately, the ICR cell is not infinite in size and its surface may initiate (catalyze) certain reactions implicating the involvement of heterogeneous chemistry on aerosols that might efficiently produce HCN and NH_3_ in Titan’s upper atmosphere.

**Figure 2:**
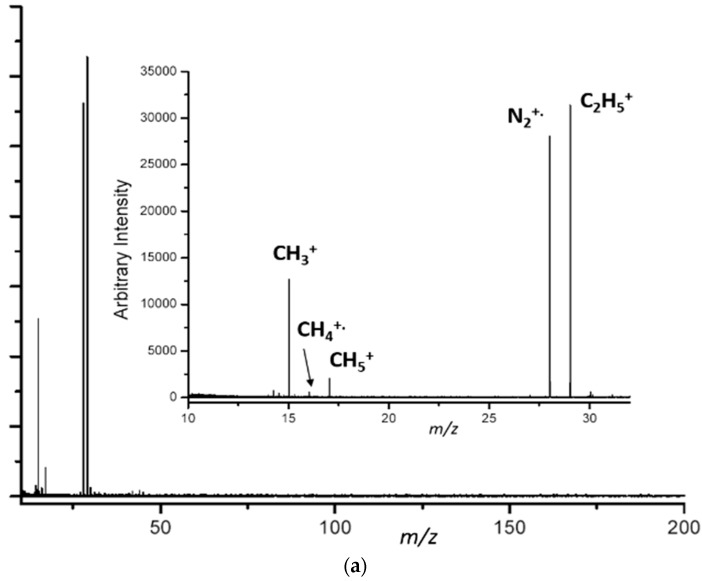

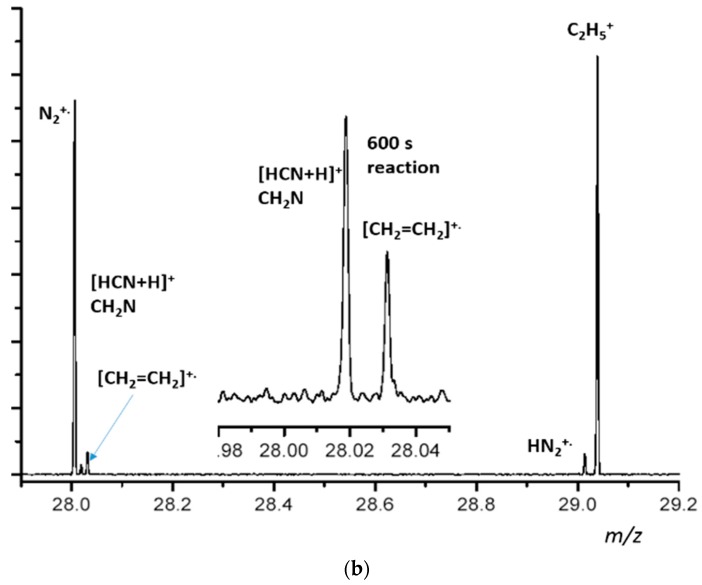
Detected reaction products obtained by a 15 s irradiation of a 95% N_2_/5% CH_4_ mixture at 5 × 10^−5^ torr after a 2 s (**a**) and 600 s (inset in (**b**)) reaction time in the ICR cell of a 4.7 T IonSpec instrument.

In parallel to the above-mentioned comprehensive studies, we also performed simple experiments in the FT-ICR cell of an IonSpec 4.7 T instrument (IonSpec, Irvine, CA, USA) where 40 eV electrons were used as the energy source (unpublished results). The electrons were generated using a regular filament mounted outside the FT-ICR cell. Again, N_2_/CH_4_ gas mixtures were leaked into the ICR cell and the electron flux was varied as well as the reaction time (*i.e.*, ions evolving time). Typical spectra are shown in Figure 2a,b.

It is expected that 40 eV electrons ionize N_2_ and CH_4_. The irradiation time of 15 s of ionization with 40 eV electrons is sufficient time to ionize these molecules efficiently. A 2 s reaction time was allowed after the electron beam was turned off so other products are also formed (within the altogether 15 + 2 = 17 s time window). Not surprisingly, products that mimic chemical ionization (CI) when CH_4_ is used as a CI gas (see, e.g., CH_5_^+^ and C_2_H_5_^+^) have also been detected. Ultrahigh resolution allows us to take a “closer look” to identify HCNH^+^ (most likely protonated HCN), N_2_^+^, and the ethylene molecular ion (CH_2_=CH_2_^+^) all with *m*/*z* 28 nominal mass. When the gas mixture was allowed to react further for 600 s after the applied 15 s electron flux, the ionized N_2_ disappeared and the ratio of CH_2_N/CH_2_CH_2_ increased (see inset in Figure 2b). Note that the intensity of C_*x*_H_*y*_ ions has been gradually increased and shifted to higher *m*/*z* masses (up to about *m*/*z* 180) and they overwhelmingly dominate the 600 s reaction time spectrum (full *m*/*z* range not shown). Ions with C_*x*_H_*y*_N_*z*_ compositions were detected at very low intensity (or not detected at all).

Unfortunately, albeit not surprisingly, similar to the Elettra experiments mentioned above [23], we also faced the problem of the involvement of the surface, as well as residual H_2_O after 10 s of reaction time, after which the H_3_O^+^ ion becomes dominant. Evidence for surface chemistry was found in the negative ion mode by the detection of Cl^−^ and Br^−^ anions (spectrum not shown) that, most likely, are sputtered products from the mesh located between the filament and the ICR cell (that functions as an “on–off” switch to control the electron beam flux in and out of the ICR cell).

In spite of the shortcomings mentioned above, we believe that using the ICR cell as a gas phase “reactor” is justified and the ions produced are informative for the very early stage of ion formation generated by either high energy photons (in the UV range) or electrons (in the tens of eV energy range). In fact, surface chemistry, albeit a different one related, e.g., to microscopic dust in the atmosphere, may play a role in the formation of C_*x*_H_*y*_N_*z*_ species that can grow to colloidal droplets responsible for the haze (non-transparent atmosphere). These droplets can rain down with liquid methane to Titan’s surface and can be exposed to additional chemical reactions, most notably hydrolysis in ammonia/ice pounds (see discussion below).

### 2.3. Structural Analysis of Laboratory “Tholins”, Analogues of Titan Aerosols by FT-MS

The first detailed ultrahigh resolution MS study on non-volatile laboratory tholin components (molecular weight > 120 Da) was published back in 2003 [24]. A homemade glow discharge (high voltage (HV)) reactor was built in the laboratory of Mark Smith (then at the University of Arizona) to generate laboratory tholin samples. This reactor was later modified and produced more pure tholin samples [25]. If methane is constantly replenished in the atmosphere, non-volatile aerosols have had billions of years to accumulate on Titan’s surface. Thus, determining the chemical composition of its constituents is crucial to better understand and predict what might happen to them on the surface. Without ultrahigh resolution, significant chemical information would have been lost. Most notably, we realized that at almost every integer *m*/*z* there are several ions that are related to a 2CH_2_/2N permutation that produce homolog series that grow and diminish with increasing *m*/*z*. (The mass difference between 2CH_2_ and 2N is 28.031300 − 28.006148 = 0.025152 Da, which is easily detectable using FT-MS even at higher *m*/*z* values.) These series become more unsaturated with increasing molecular mass. The success of this study inspired us as well as others to perform additional analysis by using ultrahigh resolution mass spectrometry [24,25]. Similar trends were observed by Pernot *et al.* [26] in a more detailed study. 

It is well known that most of the prebiotic molecules on Earth contain oxygen. Because there is almost no oxygen containing compounds in Titan’s atmosphere, the obvious question emerged: is there any way to incorporate oxygen into the C_*x*_H_*y*_N_*z*_ molecules that are initially formed in a reductive atmosphere?

One avenue is to add the N_2_/CH_4_ mixtures with small amount of CO, perform the laboratory simulations and analyze the products by ultrahigh resolution mass spectrometry. A detailed study by Hörst *et al.* was published in 2011 [27]. The laboratory simulations were performed in the PAMPRE (Production d’Aerosols en Microgravité par Plasma Réactifs) apparatus [28], producing aerosols by cold plasma activation of a gaseous N_2_/CH_4_/CO mixtures of 96.2%/2.0%/1.8% and 93.2%/5.0%/1.8%. Isotopically labeled C^18^O was used in these studies to further confirm the oxygen contribution from CO. The LTQ-Orbitrap mass spectrometer (Grenoble, France) was used to obtain the ultrahigh resolution mass spectra. Although the gas composition was significantly different from that of Titan’s upper atmosphere, the study is important and clearly shows that when simple oxygen containing compounds (such as CO) are present, complex molecules containing oxygen are definitely formed. Most interestingly, several prebiotic molecules have been detected and confirmed, including adenine, cytosine, uracil, thymine, guanine, glycine and alanine [27].

An alternative way for oxygen incorporation is hydrolysis reactions of Titan aerosols on the surface of Titan. This sounds bizarre at first because the temperature is so low (about 95–100 K) on the surface. Obviously, water is frozen but water mixed with some ammonia has a significantly lower freezing point (e.g., 29.4% ammonia/water is liquid at about 193 K). Ammonia/water ponds can, furthermore, be heated up by meteorite impacts and they can stay melted for several thousands years.

We performed some qualitative hydrolysis reactions in H_2_O and ammonia/water [29]. It was found that laboratory tholin components incorporate significant amount of oxygen within an hour at 373 K or within a few hours at 293 K. Exposure to air for several weeks also led to oxygen incorporation but the process was, as expected, much slower than in the liquid phase. In the same paper, we also proposed some characteristic fragmentation patterns using low resolution ion trap and high resolution (FT-ICR) tandem MS/MS measurements. In the positive ion mode, characteristic losses include the loss of NH_3_, HCN, C_2_H_4_, CH_3_CN, CH_2_=NH, *etc.* Another importance of this work was to demonstrate the ESI ionization with methanol:acetonitrile (1:1) solvent combination is an inert mode to generate ions from tholin components, *i.e.*, when laboratory tholins are harvested carefully and anaerobically, oxygen contamination is negligible during the ESI process and the solutions are stable for at least 1–3 days allowing us more than enough time to gather ultrahigh resolution mass spectrometry data on the samples.

More comprehensive studies on laboratory hydrolysis have been carried out by Neish *et al.* in water and 14% ammonia/water solutions at different temperatures (−20, 0, 20, and 40 °C) [30,31,32]. Kinetic curves and rate constants have been determined at those temperatures for both decay processes (*i.e.*, when C_*x*_H_*y*_N_*z*_ species decayed due to oxygen contribution) and for oxygen growth reactions (*i.e.*, the growth of C_*x*_H_*y*_N_*z*_O_*n*_ species). (Note that experiments with ^18^O labeled water clearly proved that the source of oxygen was water and not contamination from moisture in air.) Activation energies were then calculated from Arrhenius plots for several species. The activation energies were found to be in the range of 50 ± 10 kJ/mol (decay) and 60 ± 10 kJ/mol (growth) [30]. Extrapolating down to about 100 K, these values encourages us to say that hydrolysis reactions can, indeed, occur within 3000–10,000 years, which is a very short time on the planetary time scale. Again, we note for correctness, that no phase transitions (*i.e.*, freezing of melted ponds) are assumed. But even then, meteorite impact can heat up local ammonia/water environment that might cool down within a couple of days or weeks but oxygen enrichment can occur within this relatively short time even though not at full scale

**Figure 3:**
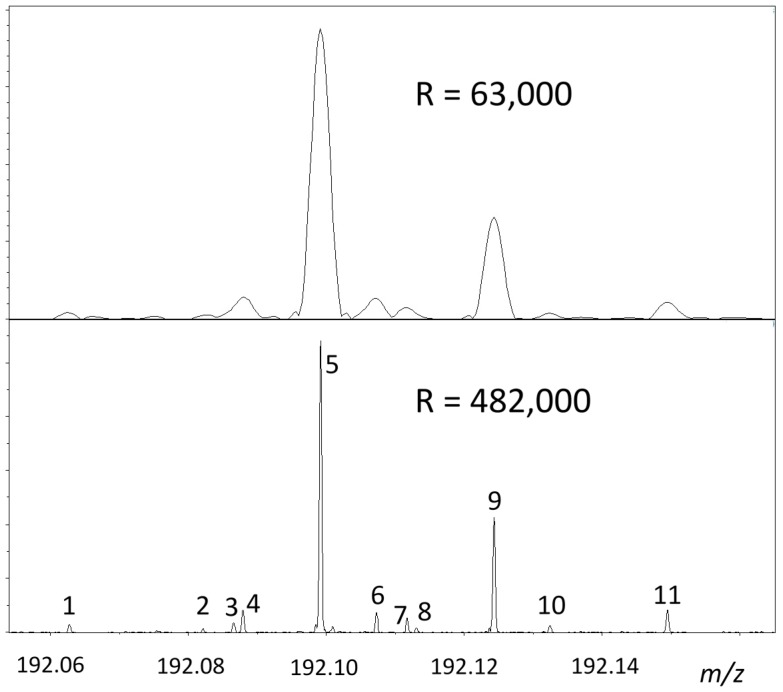
Comparison of information content obtained for LDI generated ions with the nominal mass of *m/z* 192 at two different resolutions (63,000 **upper**; 482,000 **lower** spectrum). The list of ions corresponding to the labeled peaks is collected in Table 1.

Similar to the work of Hörst *et al.* amino acids asparagine, glutamine, and histidine were detected among the hydrolysis products with 14% ammonia/water [32]. It is interesting to note here that narrow band experiments with resolution >200,000 clearly showed [31] that tholin components not only react with oxygen but also with NH_3_ as was proved by using labeled ^15^NH_3_. The power of using even higher resolution (up to almost 500,000) is demonstrated in Figure 3 and Table 1 that shows the unambiguous identification of additional species, e.g., the odd electron molecular ions for ions with the same *nominal* mass of *m/z* 192. The lower spectrum practically demonstrates an isotopic fine structure experiments that is widely used in metabolomics. Even though the bottom spectrum clearly shows ten different species, this spectrum is relatively simple compared to those obtained for species containing other mass deficient elements, such as S, P, and halogens.

Data in Table 1 show that components 5, 9, and 11 are the well-characterized species related to the above-mentioned 2CH_2_/2N replacements. In addition, it is expected to detect the ^13^C isotope peaks for species with *m/z* 191 nominal mass (3, 6, 10). This sample is the same as in used in Reference [25] (denoted as UHVT_001) so it has been stored in a −80 °C freezer for years in a parafilmed bottle but has been opened a couple of times during these years so the appearance of small oxygen containing “contaminants” is not surprising (peaks 1, and 4). What is especially interesting and not reported before is the detection of odd electron molecular ions (peaks 3 and 7). Not shown in Table 1 but the largest error for accurate mass measurement that is possible to achieve with internal calibration and with a 15 T magnet is about 20 ppb. (First, the broadband spectrum is calibrated, ion compositions are identified and confirmed, then an ion in the narrow range is used to recalibrate the isotopic fine structure spectrum.) Obviously, the top spectrum in Figure 3 with a ≈*R* = 63,000 resolution is already informative but separation of peaks 3, 4 and 7, 8 is not adequate enough.

It is important to note here that the spectra shown in Figure 3 have been obtained on our Bruker SolarixR 15T FT-ICR instrument (Ohio State University, OH, USA) with an alternative ionization technique, laser desorption ionization (LDI). We first realized the effectiveness of LDI for laboratory tholin ionization back in 2005 [29] by generating LDI ions up to *m*/*z* 1000. That time LDI with FT-ICR capability was not available in our laboratory so only a time-of-flight (TOF) instrument (a Bruker Ultraflex III MALDI TOF-TOF, University of Arizona, Tucson, AZ, USA) was used. The resolution of TOF instruments (usually <50,000) is not always adequate to detect all “isobaric” ions, although they still can provide useful results just as demonstrated in [33] for tholin samples or de Marcellus *et al.* for aldehyde and carbohydrate molecules evolution in laboratory simulations by GC-TOF mass spectrometry [34].

Most of the studies mentioned above focused on positively charged ions (except with [33] that presented results for negatively charged ions). Recently, we performed a comparative study for positively and negatively charged ions by using both ESI and LDI ionization methods [25]. Characteristic differences have been observed: more saturated positively charged ions represent mostly amines and imines, meanwhile negatively charged ions are more unsaturated and represent compounds with nitrile (cyano) functionality (CN groups). In some cases, exotic C_*x*_N_*z*_ ions are observed in the negative LDI spectra, such as the C_10_N_5_^−^ anion that is most presumably a pentacyano cyclopentadienyl anion. Tandem MS/MS fragmentation confirmed the cyano functionality by losing HCN as a neutral molecule from several ions. Tandem MS/MS fragmentation and quantum chemical calculations suggested the formation of ion-molecule complexes, with a common anion of C_2_N_3_^−^. (This ion at *m*/*z* 66 was first mentioned and studied in detail by Carrasco *et al.* [33].) Nevertheless, further experimental and theoretical studies are necessary to confirm the existence of stable ion-molecule complexes.

**Figure 4:**
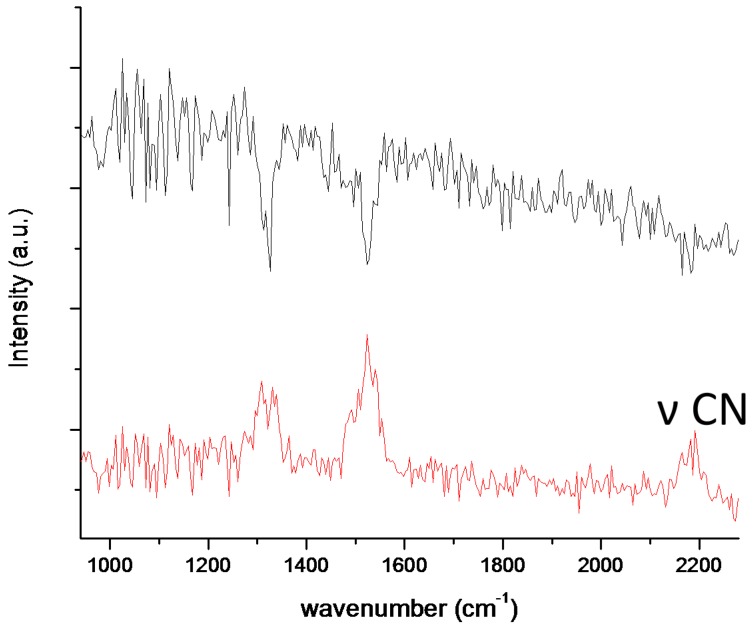
IRMPD action spectra of the anion C_4_H_3_N_4_^−^. The **upper** trace represents the trapped precursor ion *m/z* 117; and the **lower** trace represents all fragments of *m/z* 117 as a function of the IR radiation wavenumber. The CN stretching band is detected around 2180 cm^−1^.

It is clear from the experimental and theoretical studies mentioned above that structural isomers do exist. Thus, even with the ultrahigh resolution power of FT-MS, isotope distinction is not always possible. Other than theoretical calculations, there are three different mass spectrometry related experiments that can be used to distinguish isomers: (i) ion mobility; (ii) gas-phase HDX; and (iii) variable wavelength infrared multiphoton dissociated spectroscopy (IRMPD). Although we have performed some ion mobility and HDX experiments in our laboratories, the result for laboratory tholins are not conclusive enough to be publishable. On the other hand, we successfully used IRMPD action spectroscopy in the CLIO laser facility [35] to obtain information on functional groups. The evaluation of the data and theoretical calculations are in progress and the results will be published elsewhere. For illustration, in Figure 4 we show one IRMPD action spectrum that was obtained for a C_4_H_3_N_4_^−^ anion (that we assume is an isomer (or isomers) of “deprotonated tetracyanide”). To our best knowledge, this is the first time a nitrile (CN stretching) band was detected at around 2180 cm^−1^ in the CLIO facility. This is an important result because it provides proof for the existence of –CN groups in highly unsaturated anions.

## 3. Materials and Methods

The experimental details are quite diversified so we ask the readers to check the cited references below for details. Nevertheless, for guidance and easier reading, we summarize briefly the main features regarding model studies and instrumentation used in our laboratories. The individual references will be shown in the Results and Discussion Section.

Laboratory tholin samples were prepared by exposing a mixture of 2%–5% methane and 98%–95% nitrogen to a radio frequency electrical discharge under slow flow (6 atm L·h^−1^ and a pressure of 10^−2^ bar) at a temperature of 195 K in a recently designed ultrahigh vacuum (airtight) reaction chamber. The tholins were collected anaerobically in a glove box and stored in carefully sealed vials to avoid contamination so that tholin samples were produced with high purity (*i.e.*, only a trace amount of oxygenated species are detected, see discussion below).

The tholin samples were directly deposited on a conventional (stainless steel) matrix assisted laser desorption/ionization (MALDI) plate for laser desorption ionization (LDI) measurements that were carried out on a 9.4 T Bruker Daltonics Apex-Ultra FT-ICR instrument (at the University of Arizona, Tucson, AZ, USA) and a Bruker 15T SolariXR instrument (at the Ohio State University, Columbus, OH, USA). A Yag:Nd laser (352 nm) was used with variable laser power to reduce the possibility of laser induced fragmentation. Both FT-ICR instruments are equipped with a dual ESI/MALDI source so electrospray ionization (ESI) was also used for comparison. A Thermo Fisher Scientific LTQ-Orbitrap-XL instrument (University of Grenoble Alpes, Grenoble, France) was also used to perform comparative ultrahigh resolution MS and tandem MS/MS experiments. For electrospray ionizations, the tholin samples were dissolved in methanol and methanol:acetonitrile (1:1) with a ≈1–10 micromolar concentration. In all experiments, the instrument conditions were tuned and optimized for the detection of both positively and negatively charged ions in the mass range of *m/z* 50–400. Some experiments with electron ionization were also performed in the FT-ICR cell of a 4.7 T IonSpec instrument. Infrared multiphoton dissociation (IRMPD) action spectroscopy measurements were carried out at the Centre Laser Infrarouge d’Orsay (CLIO) facility [35] (Université Paris-Sud, Orsay, France). IRMPD action spectroscopy measures the trapped ion fragmentation efficiency as a function of the IR photons’ wavenumber, *i.e.*, not the IR light absorption is recorded but the “action” (fragmentation) of the ion. 

Before we show and discuss some recent results, we summarize briefly what kind of information can be obtained from ultrahigh resolution mass spectrometry (UHRMS) experiments and how data processing can simplify and make the information content more informative.

The ultrahigh resolution capability of Orbitrap (up to about 450,000, see Reference [36]) and ICR instruments (up to about 20,000,000 with a 21T magnet [37] and with the recently developed dynamically harmonized cell [38]) allow us:

(i) To separate and determine major and minor components in complex mixtures, *i.e.*, significantly improve the chemical information. Without ultrahigh resolution, this information would be lost.
(ii) To perform *accurate mass* measurements (*i.e.*, chemical composition determination) within <100 ppb error with internal calibration. Isotope fine structures [39] can be determined so that “isobaric” elements (e.g., ^32^S (31.972071) and 2 O (31.989829)) are distinguished and used to confirm/eliminate certain chemical compositions. It has been shown that a mass resolution of 5,000,000 and mass accuracy of 100 ppb are sufficient to unambiguously ascribe the correct elemental composition for ions containing C, H, O, N, and S up to 500 Da [40].
(iii) To determine fragmentation pattern of several precursor ions, *i.e.*, speeding up structural determination. Practically, this means data independent acquisitions (DIA) [41] that are widely used for complex metabolomics samples. Knowing the chemical compositions and characteristic fragments, e.g., loss of H_2_O, NH_3_, HCN, CH_3_CN, *etc.*, one can easily generate a theoretical fragmentation matrix that can be compared to experimentally observed fragments to reveal structural features of individual components.

Instead of tabulating literally hundreds of chemical compositions, simple but informative representations, such as the Kendrick mass diagrams [42] (or mass defect *vs.* exact mass (MDvEM) plots [43]) or the (modified) van Krevelen diagrams [44,45] are commonly used for data interpretation. These diagrams are especially helpful in determining homolog series, such as molecules differing by CH_2_ units, and chemical modifications, e.g., when 2 CH_2_ units are replaced by “isobaric” (but, by FT-MS, easily distinguishable) 2 nitrogens. A total mass difference statistic algorithm was recently developed for the improved analysis of FT-ICR data obtained for natural organic matter that can be applied to organic samples of astrobiological importance [46].

## 4. Conclusions

Research on the formation of prebiotic, small organic molecules is a prerequisite to any further investigation about the origin of life in the Universe. Although we can now see far away and we can detect objects (galaxies) in the far remote corner of the observable Universe, we are very much bound to Earth and our Solar System to obtain detailed information on the existence (or absence) of prebiotic molecules. Planets have been detected around nearby stars (within about 100 light years) and improving instrumentation allows us to anticipate the possibility to detect “signatures of life” in the form of small organic molecules in planetary atmospheres. Nevertheless, detailed information on a wide variety of organic structures is not expected to be available for at least a couple of decades. For the time being, it is completely justified to study Solar System objects, such as meteoritic materials. Processes in the reductive atmosphere of the relatively close Titan need to be better understood for general chemical curiosity. The species formed in a reductive gas mixture under controlled laboratory conditions may provide insight for the origin of small, C, H, and N containing organic molecules.

The degree of information harvestable and its reliability strongly depend on the analysis methods and instrumentation. In this regard, ultrahigh resolution mass spectrometry (URHMS) is the best available method to obtain information on *individual* components of very *complex mixtures.* Nature tends to produce complex mixtures and not pure samples thus it is an obligation to use the best method that is available to unveil (certain) secrets of Nature.

We do hope that the illustrative examples shown above convince the readers about the importance, usefulness, reliability and necessity of using UHRMS in astrobiological research. Organic contents of meteorites, the products of atmospheric processes, and their further reaction (hydrolysis) products, can now be followed with incredible resolution (>500,000) and mass accuracy (<1 ppm). Isomeric structures can be distinguished by coupling UHRMS with additional techniques, such as IRMPD action spectroscopy, gas-phase HDX, and ion mobility.

Although the results obtained in laboratory environment on Earth are promising and revealing more and more structural details, *extrapolation* to other objects will always be risky. We do know a lot about the components and structure of laboratory-made tholins but we cannot be absolutely sure that these molecules are *really* there in the atmosphere and/or surface of Titan. Nevertheless, we strongly believe that the laboratory results will inspire the design of *ultrahigh resolution* mass analyzers that will, eventually, be sent to space, planets, moons, meteorites and comets. In fact, some of these design projects are in progress (multibounce TOF selected for future NASA mission at Europa [47] and Cosmorbitrap project to adapt the Orbitrap concept into a space borne instrument [48,49,50,51,52,53]). The technical challenges are enormous but physics allows such dreams that we the human species make to become reality.

## Figures and Tables

**Table 1:** Chemical compositions of the ions with nominal mass of *m/z* 192 at about 500,000 resolution (bottom spectrum in Figure 3).

Peak	Ionic Composition *	Calculated *m/z*	Measured *m/z*
1	C_6_H_6_N_7_O	192.06283	192.06282
2	C_6_H_7_N_8_ (^13^C)	192.08217	192.08218
3	C_6_H_8_N_8_	192.08664	192.08657
4	C_8_H_10_N_5_O	192.08799	192.08799
5	C_7_H_10_N_7_ *	192.09922	192.09922
6	C_8_H_11_N_6_ (^13^C)	192.10733	192.10732
7	C_8_H_12_N_6_	192.11180	192.11179
8	C_10_H_14_N_3_O	192.11314	192.11311
9	C_9_H_14_N_5_	192.12437	192.12437
10	C_10_H_15_N_4_ (^13^C)	192.13248	192.13248
11	C_11_H_18_N_3_	192.14952	192.14952

* denotes the ion used for internal calibration and ^13^C indicates that one carbon atom is a ^13^C isotope.
